# Supplementary material for: Analysing Power Relations among Older Norwegian Patients and Spanish Migrant Nurses in Home Nursing Care: A Critical Discourse Analysis Approach from a Transcultural Perspective
Source: Healthcare (Basel). 2023 Apr 29;11(9):1282. doi: 10.3390/healthcare11091282 (PMC10178409; doi:10.3390/healthcare11091282)
Supplement: Supplementary file 1 [file healthcare-11-01282-s001.zip › healthcare-2287073-supplementary/230301_Table-S1-COREQ-checklist.pdf]

Table S1. Report using the COREQ checklist for reporting qualitative research.

Domain 1: Research team and reflexivity	Description
**Personal Characteristics**	
1. Interviewer/facilitator<br>Which author/s conducted the interview or focus group?	PMA conducted all the interviews.
2. Credentials<br>What were the researcher's credentials?	PMA was a PhD student and MSc in Health sciences research. MRR, PVP, and SLQ were PhDs.
3. Occupation<br>What was their occupation at the time of the study?	PMA was working as a Research Fellow at the University of Córdoba (UCO). MRR and SLQ were working as full professors at UCO. PVP was working as an assistant professor at UCO.
4. Gender<br>Was the researcher male or female	PMA, MRR, PVP, and SLQ are male.
5. Experience and training<br>What experience or training did the researcher have?	PMA had experience in qualitative research from previous works. He received a Master's degree in Health sciences research from the University of Jaén (UJA), Spain. In addition, he underwent formal PhD education in qualitative research and gave teaching lectures during his fellowship.
**Relationship with participants**	
6. Relationship established<br>Was a relationship established prior to study commencement?	There was no previous relationship with any interviewee since the interviewer of the research team knew them at the time of data collection. PMA had a prior relationship with the rest of the group: MRR and SLQ were their doctoral thesis supervisors; PVP was a colleague from the department.
7. Participant knowledge of the interviewer<br>What did the participants know about the researcher? e.g. personal goals, reasons for doing the research	PMA informed the participants that the research project belonged to a thesis project of PMA. The leading researcher told Norwegian older patients and Spanish migrant nurses his purpose with the study. When the participants asked questions about the project, they were answered by both researchers.

8. Interviewer characteristics<br>What characteristics were reported about the interviewer/facilitator? e.g. Bias, assumptions, reasons and interests in the research topic	The principal interest of PMA in the topic was based on his desire to focus its thesis project and future research on situations of social injustice, imbalance of power, and possible discrimination in care contexts in the primary care setting.
**Domain 2: study design**	
**Theoretical framework**	
9. Methodological orientation and Theory<br>What methodological orientation was stated to underpin the study? e.g. grounded theory, discourse analysis, ethnography, phenomenology, content analysis	The research paradigm for this study was hermeneutic with a critical perspective. Using an interpretative methodology, researchers could dive into the reality of other individuals. Using a critical discourse analysis (CDA) perspective, the researchers had a reflective approach to possible social injustice and discrimination situations to locate care failures and highlight areas for improvement in the care from health services. Having a transcultural perspective, the researchers could have an insight into how the cultural shock affected the care experience of Spanish migrant nurses; besides, they were culturally competent professionals. This study analyses the power relationships between Norwegian older patients living alone and Spanish migrant nurses attending them at home. We followed Crist & Tanner´s hermeneutical considerations, alongside linguistic analysis regarding CDA, to understand participants´ interpretations of the phenomena studied.
**Participant selection**	
10. Sampling<br>How were participants selected?<br>e.g. purposive, convenience, consecutive, snowball	The article explained the sampling method, and all approached Norwegian older patients, and Spanish migrant nurses agreed to participate.
11. Method of approach<br>How were participants approached?<br>e.g. face-to-face, telephone, mail, email	All Norwegian older patients were approached face-to-face with help from nurse care managers and referral nurses who reached them. In the case of Spanish migrant nurses, snowball sampling was employed through email.

12. Sample size<br>How many participants were in the study	There were fifteen semi-structured interviews; eleven were with older Norwegian patients, and four were with Spanish migrant nurses.
13. Non-participation<br>How many people refused to participate or dropped out? Reasons?	None of the participants who were asked to participate refused to be part of the study or withdrew from it at any time.
**Setting**	
14. Setting of data collection<br>Where was the data collected? e.g. home, clinic, workplace	The interviews took place at the participant's preferred location. This location was their home for Norwegian older patients. Zoom platform via videoconference with Spanish migrant nurses.
15. Presence of non-participants<br>Was anyone else present besides the participants and researchers?	Only the participant and the researcher were present in all the interviews.
16. Description of sample<br>What are the important characteristics of the sample? e.g. demographic data, date	All participants´ characteristics are described in Tables 2 and 3.
**Data collection**	
17. Interview guide<br>Were questions, prompts, guides provided by the authors? Was it pilot tested?	The authors provided the interview scripts as supplementary files to this article. The list of topics was adjusted to each situation´s uniqueness throughout the research interview phase.
18. Repeat interviews<br>Were repeat interviews carried out?<br>If yes, how many	Repeated interviews with the participants were not conducted. As for the Norwegian older patients, this was due to their multimorbidity and because many reported their state of fatigue concerning the realisation of interviews. As for the Spanish migrant nurses, this was due to their difficulties in joining again.

19. Audio/visual recording<br>Did the research use audio or visual recording to collect the data?	All interviews were audio recorded with the participant's verbal and written consent. The recordings were stored on a PMA computer because he was responsible for data analysis. Only he had access to this data.
20. Field notes<br>Were field notes made during and/or after the interview or focus group?	The audio recording was accompanied by a hermeneutical diary which included observations and impressions that were not recorded, such as the non-verbal communication of the participant. It contained reflections through a self-hermeneutic process during the study as well. Field notes were used in the analysis of the results afterwards.
21. Duration<br>What was the duration of the interviews or focus group?	The time of the semi-structured interviews was approximately 50 minutes on average.
22. Data saturation<br>Was data saturation discussed?	Data saturation was discussed with the research team.
23. Transcripts returned<br>Were transcripts returned to participants for comment and/or correction?	Due to various reasons (such as the limitations in the reading of most of the older patients due to medical or literacy issues; a state of exhaustion after the completion of the interviews), the transcripts were not returned for comments or feedback. The transcriptions were not returned to the migrant nurses because of time unavailability.
**Domain 3: analysis and findings**	
**Data analysis**	
24. Number of data coders<br>How many data coders coded the data?	PMA performed the critical discourse analysis. MRR supervised the research alongside SLQ.

25. Description of the coding tree<br>Did authors provide a description of the coding tree?	No coding tree was used. The themes were derived from the data, following Crist & Tanner´s hermeneutical considerations alongside a linguistic analysis in the data analysis phase. The authors provided narrative and visual development of this process in the article.
26. Derivation of themes<br>Were themes identified in advance or derived from the data	The themes were derived from the data and were discussed and agreed on by all the authors.
27. Software<br>What software, if applicable, was used to manage the data?	SimpleMind Pro was the software tool for managing the data visually.
28. Participant checking<br>Did participants provide feedback on the findings?	Due to several reasons, as explained at number 23, there was no feedback from the participants on our findings after the interviews. During the interviews, the interviewers repeated and summarised the participants´ answers to ask for clarification and confirmation of the researcher's interpretation of the answers. At the end of the interview, the researcher summarised the content to ensure the researcher understood the main content correctly. At the end of the interview, the researcher also asked the older patients and migrant nurses a final question about possible comments they wished to make regarding what had been said and potential topics not raised through the conversation but that they wanted to make explicit.
**Reporting**	
29. Quotations presented<br>Were participant quotations presented to illustrate the themes/findings? Was each quotation identified? e.g. participant number	The results section is illustrated with speech acts as quotes from the participants. Each quote is identified with a participant number.
30. Data and findings consistent<br>Was there consistency between the data presented and the findings?	According to our assumption, the data presented in the study and the results that emerge from them are consistent.

31. Clarity of major themes<br>Were major themes clearly presented in the findings?	The major themes are present in the results section of our article. Each theme is assigned a different heading.
32. Clarity of minor themes<br>Is there a description of diverse cases or discussion of minor themes?	The minor subthemes are described along with the major themes, accompanied by specific quotes for each one.
