# Supplementary material for: Analysing Power Relations among Older Norwegian Patients and Spanish Migrant Nurses in Home Nursing Care: A Critical Discourse Analysis Approach from a Transcultural Perspective
Source: Healthcare (Basel). 2023 Apr 29;11(9):1282. doi: 10.3390/healthcare11091282 (PMC10178409; doi:10.3390/healthcare11091282)
Supplement: Supplementary file 1 [file healthcare-11-01282-s001.zip › healthcare-2287073-supplementary/230301_Table-S2-reflexivity-criteria.pdf]

Table S2. Reflexive questions following Joseph et al. [50].

Reflexive questions	Researcher responses
**Domain 1: Sensitivity**	
What are the participants' and the community's experience of qualitative research?	Participants had no previous experience in taking part in qualitative studies.
How does the gender and age of participants define the method of communication?	The interviewer was a man of 28 years old. Concerning Norwegian patients, all were women with a mean age of 83. Concerning Spanish migrant nurses, all were women with a mean age of 26. The communication method was also influenced by the language used: the native language in the case of interviews with migrant nurses and the language learned in the case of older patients. This, added to the age difference concerning older patients, determined the communication method more directly and compared to migrant nurses, with whom the interviewer felt more comfortable and with a more accessible language from the point of generational and idiomatic view.
How intrusive are the research topic and research questions?	The interview questions were direct about: whether they were told to choose something, if they felt heard or if they participated in something, for older patients; if they gave older patients something to choose, if they listened to older patients, if they encouraged to participate, and how the experience in Norway affected them in their personal/ professional plane, in the case of migrant nurses. However, the research team took care of the script so that the questions were polite.
Is the research topic related to a taboo subject or issues normally only discussed within the household?	No

Are there areas in the research community that are restricted or inaccessible to researchers due to their socio-demographic characteristics?	No
What is the research community's perception of the research topic?	The study of interactions in care between nurses and patients from different cultures is perceived as necessary and current by the scientific literature, as well as being key in an increasingly globalised world where health needs must be urgently met.
Where are the points of potential sensitivity?	The points of potential sensitivity were the explicit examples that patients and nurses used based on their vital experiences about situations where quality care was not being carried out concerning the study phenomena. In other moments they identified a manifest power imbalance with negative consequences in the nurse-patient relationship.
Does the researcher have the set of skills needed to show cultural sensitivity?	The leading researcher demonstrated cultural sensitivity skills because at no time did the patients or nurses refuse to answer any questions or give examples. The leading researcher knew the Norwegian language and had worked as a home nurse in Norway for two years. He knew the work and cultural context he found himself in during the interviews with Norwegian older people —the same with the Spanish migrant nurses.
What are the participants' expectations from the researcher and the study?	The Norwegian older patients expected the research to make their situation visible and to be heard. The Spanish migrant nurses expected the study to share their experiences and make their work and personal context visible in a different culture.
**Domain 2: Vulnerability**	

Are participants able to make an informed decision on whether to consent to take part?	Yes
What is the protocol for community entry; does it require permission from a gatekeeper to approach potential participants?	In the case of Spanish migrant nurses, it was not necessary. In the case of Norwegian older patients, it was required to approach the supervising nurses first to approach potential participants.
What is appropriate for participant reimbursement?	There were no costs that were reimbursable in this study.
Are participants at risk of coercion or exploitation?	No
Which characteristics of the participants expose them to risk of exploitation?	In the case of patients, having advanced age and living alone. In the case of migrant nurses, their situation of cultural vulnerability.
Does the researcher's identity pose or worsen physical and emotional risk?	No
What makes researching these social groups different to researching others?	This research precisely bases its importance on the relationship between two opposing social groups, finding differences in age, work context, and cultural context. Studying these social groups and their interaction makes a difference in the conception of care situations in which power relations in a different cultural environment are not considered.
How might participants' vulnerabilities prevent researchers from accessing research sites and undertaking their research?	In the case of Norwegian older patients, living alone and conducting the interviews in their homes could be limiting. However, the leading researcher gave the choice of where the interview would occur at all times to alleviate this potential consequence. All the older adults could decide on the interview place, date and time slot. In the case of Spanish migrant nurses, special consideration was also

	given to their low availability due to labour issues, so we perfectly adapted to their needs.
How can the research team ensure that their research processes will not further marginalize the vulnerable?	The research team implemented good practices to engage in culturally sensitive interview practices that could be seen in the article Data collection section.
Is the researcher also at risk?	No
What safety measures need to be taken before embarking on the study for the researcher and participants?	We carry out measures such as informing the participants in detail about the study and objectives and ensuring the confidentiality of the data obtained. We also request informed consent by signature and bioethical permission from the respective competent institutions in Spain and Norway. We received approval for our study before the data collection phase.
**Domain 3: Cultural identity**	
Is the researcher aware of their own cultural beliefs and how they might influence the interpretation of the participants' responses?	Yes. The leading researcher assumed sociocultural elements that shaped the meetings and reflected them in a hermeneutical diary.
Is the researcher knowledgeable about how culture and religion can affect participants' participation in the research?	Yes [see response above].
Is the researcher knowledgeable about how cultural norms may influence communication, for example, decision making roles (should be mutual between researcher and participant), eye contact, interpersonal space, use of gestures, asking and responding to questions, and use of humor?	Yes. The leading researcher was knowledgeable about proxemics, kinesics, body language, and cultural norms of the encounters.

What facets of the researcher's and participants' identities are most significant in the ongoing research?	The most determining facets of identity in this study were the self-concept of the older patient, in the case of the Norwegian older adults, and the self-concept of the outsider, in the case of the Spanish migrant nurses. In the case of the researcher who carried out the interviews and the critical discourse analysis, the outsider researcher-nurse culturally adapted to the context of the present study.
How do the participants relate to the researcher's identity?	In the case of older Norwegian patients, there was an empathic listening relationship by recognising the researcher as a professional interested in their situation and cultural context. In the case of migrant Spanish nurses, a fluid and empathetic relationship was due to sharing professional, cultural and personal grounding with the researcher.
Are there enablers or helpers in the research setting and, if so, how can the researchers identify them?	No
Does the researcher's identity make this research a more sensitive topic, leading to a discussion that could upset the participants?	No
How does the researcher's identity influence the cultural expectation of participants and the host community?	The researcher's culturally distinct identity concerning Norwegian older people probably defined their expectations. The older patients emphasised the situations experienced to the researcher so that he was fully aware of what it was generating in the discourses. The host community and the participants represented a cultural challenge for the researcher.
Does the research team have the necessary knowledge, skills, and tools for reflexivity?	Yes. Taking the Campinha-Bacote model as a reference, self-hermeneutics was exercised as a reflective introspection.

Does the researcher have the skill to adapt their communication style to effectively interact with people who communicate in ways that are different from their own?	Yes. The leading researcher had previously dealt with Norwegian older people during his practice as a home nurse in Norway, so he cultivated his communication skills in that cultural context to provide care and communicate effectively.
Does the researcher have the skill to consistently act in ways that demonstrate respect for the culture and beliefs of others?	Yes. Transcultural nursing is based on a respect for others´ cultures and identities that the leading researcher cultivated while working as a nurse in Norway.
How can the researcher gain acceptance within the community and from the participants?	We gain acceptance through a gradual approach to the participants and by carrying out full transparency and accessibility regarding the study and the ethical issues associated with it. On the other hand, we always proposed maintaining a relationship of warmth and empathy towards the beliefs and culture of the participants.
Do the researchers have adequate information from interpreters and community members about the study community including appropriate social routines/practices?	The leading researcher was assisted by Norwegian research nurses who were fully aware of the Norwegian nursing care context, codes and culture.
What are the researcher's biases and assumptions about the community?	Preconceived ideas about the community of older patients living alone and receiving nursing home services were that they probably suffered from loneliness and some of their unmet needs.
