# Supplementary material for: Analysing Power Relations among Older Norwegian Patients and Spanish Migrant Nurses in Home Nursing Care: A Critical Discourse Analysis Approach from a Transcultural Perspective
Source: Healthcare (Basel). 2023 Apr 29;11(9):1282. doi: 10.3390/healthcare11091282 (PMC10178409; doi:10.3390/healthcare11091282)
Supplement: Supplementary file 1 [file healthcare-11-01282-s001.zip › healthcare-2287073-supplementary/230301_Table-S3-Results-ACDT.pdf]

Table S3. Major themes, minor themes and informative excerpts of CDA from the research corpus.

<table>
<tr><th colspan="2">Major theme 1: Interferences in communication</th></tr>
<tr><th>Minor theme 1: Difficulty with expressing preferences</th><th>Minor theme 2: Clear theory, unclear practice</th></tr>
<tr><th>Norwegian older patients</th><th>Spanish migrant nurses</th></tr>
<tr><td>(1) Sometimes, yes. But not much. But they can have a lot to do, and it´s not always the worst with me. There may be someone worse [shy laugh] (no. 1).
(2) If I need something, I ask for it. It's not hard to talk to them, either. But you know that some want to, and some don’t. That can be seen (no. 1).
(3) Maybe they could talk a little more with me… [laughs] (no. 2).
(4) I don't ask for anything; I count on them to do what they must do (no. 3).
(5) Not all who come are the same, some talk a little with me, and others do what they have to do (no. 4).
(6) If I'm sunk, yes, if I'm in the process of sinking, I don't usually comment on it (no. 7).
(7) I'm not a person who talks too much. I prefer to keep to myself. But I arrange it alone. I arrange it (no. 7).
(8) I've never been used to that; I’ve always been to figure out life for myself. So it's hard... (no. 8).
(9) It's easier to tell them depending on who, but I get anxious when I see certain things happen. Everyone has the right to express themselves and give their opinion. But I have difficulties expressing myself, yes (no. 8).
(10) Once, I got up and had to lie down again because I was dizzy. And I was in the hospital, and I had a drop in blood pressure. And the nurses knew that. But they told me, “Bah, that passes immediately” (no. 8).
(11) If it [what she asks] ends up being done? No. I have told them several times to come a little earlier, but there must be something they don't like, so I feel a bit guilty [laughs] (no. 8).
(12) It happens that I usually say what I think, and before I even signed the papers that said I would receive specific help, they have not followed it (no. 9).
(13) I could only say that the afternoons get very long in winter, especially when you feel alone (no. 10).</td><td>(14) Asking them what did they think of the service? I don't know, no, no... (no. 12).
(15) To me, they tell complaints, personally [laughs]. It's like that when you go their home, people complain, and the good things are valued many times less than the bad ones, I also tell you (no. 12).
(16) I think that, after all, when you go to a house and have an estimated time, you have to say, "I'm here for this; do you need anything else?" But of course, they will always need something more (no. 12).
(17) I think that if you are open, it will cost you less; if you are shyer, it will cost you more to communicate. If you think about it and are empathic: if a person comes to your house because you can no longer do things as you could before... It is challenging to communicate. Many things are not said, I think (no. 12).
(18) Despite feeling limited by the Norwegian language, I have had the opportunity to sit down and listen to a patient for half an hour about what they wants, what they doesn´t like, what they needs, in short... How can I make them feel comfortable? That´s not so easy in Spain (no. 13).
(19) It is essential to listen to patients; for example, there are those with depression who sit on the sofa and say they are not sociable. They need to be heard (no. 13).
(20) I think it depends on the personality of each one. Because there are people who don´t want to adapt and do everything as they wish (no. 13).
(21) I guess it depends on what it is. I had a case of an older person who did not want to go to a hospital, and we could not give her optimal care at home. Her daughter also wanted to admit her to a hospital. In the end, the older patient had bilateral pneumonia. And she ended up dying there. So I don't know to what extent the patient's opinion should be respected or when their preferences should be followed. In those cases, I think about care. If it is unrealistic to follow her wishes, and I cannot guarantee care, they cannot be in an unsafe environment (no. 14).
(22) If they have to complain, they do it a lot. They also tell you what they like and don't like (no. 15).</td></tr>
<tr><th colspan="2">Major theme 2: Asymmetry in decision-making</th></tr>
</table>

**Minor theme 3: Not deciding at all**	**Minor theme 4: Real situations or mirages?**
**Norwegian older patients**	**Spanish migrant nurses**
(23) Those who work there [referring to the nursing staff who work in the office and organise the visits] decide. And it seems that it's all right, most of the time (no. 1).<br>(24) No. I can't remember any [actual situation of decision-making] (no. 1).<br>(25) Yeah, no… [pause] [laughs] I don't know [laughs]. I haven't had any situations yet, but you never know (no. 2).<br>(26) They are the ones who carry all that. It's fine the way it is (no. 3).<br>(27) I have nothing to tell… They do what they do, full stop (no. 3).<br>(28) No, I only receive visits and medicines. I have not had any possibility about what you say [to be able to decide on something] (no. 4).<br>(29) [Pause] No, nothing special (no. 6).<br>(30) Decide? No, I couldn't [Pause]. For example, I don't really like taking a shower in the morning. I do not do it. That's why I can smell a little sweat from time to time. That's why they go after me sometimes. “You have to shower” [he repeats it several times]. And then I'm… [he gestures of being fed up, holding his head] (no. 7).<br>(31) I could gladly think about it. Decide here and there a little like today; I feel strongly about that or not (no. 8).<br>(32) No, I don't choose anything; here comes the one that had to come... (no. 9)<br>(33) No... I don't see that there have been any such situations, really (no. 11).	(34) In the case of certain conflictive patients, you have to stand up. For example, if they want certain things done their way (no. 12).<br>(35) As simple as asking them, "hey, what do you want me to make you for breakfast?" Well, some colleagues do not. That seems... They´re losing their independence; at least, you have to ask them (no. 12).<br>(36) Sometimes, we are a bit "the bosses" who decide what is done or is not done. If, for example, I see that something beneficial can be done and is suitable for the patient, I try to do that (no. 13).<br>(37) Certain patients sometimes say they don´t want to take their medication today. So, I don't try to force them but to convince them why they should take medicine and why it is good for them. But the decision always comes from the patients (no. 13).<br>(38) As for the treatments, patients usually do what the doctor tells them, regardless of whether it is positive for older people. They often don´t make a clear decision due to ignorance unless it is explained to them. Other times they do not intervene because of family issues when the family dismisses the patients as older, so they decide for them. That is something that I find sad (no. 14).<br>(39) Here in Norway, the opinion of older patients is taken into account very much. I even consider that sometimes too much [laughs] because once I had a patient with dementia who was not in all his faculties and made decisions that affected the assistance and quality of health of that person, being validated (no. 15).
**Major theme 3: Heterogeneous participation and its influential elements**	
**Minor theme 5: Small and irregular performances**	**Minor theme 6: A well-oriented participation is vital**
**Norwegian older patients**	**Spanish migrant nurses**
(40) No. Because I prefer to rest a bit (no. 3).<br>(41) Well, I usually say when it´s a bit left until the nebulisers run out. I have also washed off the nebuliser mask several times for the next time. No one has asked me to do it (no. 4).<br>(42) There have been times when it has not been necessary to irrigate, and I have said it before (no. 5).<br>(43) I also usually leave the material prepared for when they come to irrigate my urinary catheter (no. 5).<br>(44) No… I don't know what to answer [laughs] (no. 6).	(50) I think that some older people do participate. As far as possible, when I go to a house, I try to get them to do what they can because we are here to help (no. 12).<br>(51) If they can do something, let them do it. After all, it is their daily training. Because if not, they end up deteriorating more. They stop being people (no. 12).<br>(52) In nursing matters, such as intravenous treatments, these are things that you are the one to do. But for example, if we are talking about personal hygiene, something as simple as saying, "I'll go with you to the bathroom, and you clean up there, and I'll help you below." (no. 12).

(45) Let's say it's Saturday or Sunday, so I'm not here around 8-9 a.m., and then I can stay in bed until 9:30 or 10 a.m. Then I let them know when I get up so they can come (no. 7).<br>(46) [Participation] means absolutely everything. Because you don't become something like... You should avoid being behind them all the time so you can do things too (no. 8).<br>(47) Not because I'm thinking all the time... I think about this, I think about the other... I've been alone for a long time... So no... I sleep poorly for a couple of hours, and then I come and sit on the couch (no. 9).<br>(48) I think I could do more than I do. They don't let me get up and cut bread; they don't let me mop the floor… And those are the kinds of things I'd like to do… Uh… because I'm still a person… I can knit a little… like this. And it is partly the nurses who decide. They don't come in much more than to give me insulin… (no. 11).<br>(49) I would like to try a little more to do more things alone (no. 11).	(53) I think that the nurse is the one who guides the older patients when we go to their homes; if they are not told what they can do, time is saved, but you don't let them participate. But that is not fair, even if it takes longer. The problem is time (no. 12).<br>(54) We always try to involve the patients as much as possible. Our routine is to explain to the patient what needs to be done and ask their opinion whenever they are oriented; if not, we ask the family's opinion (no. 13).<br>(55) Participation is paramount as long as they are receptive. That's why it's essential to ask (no. 13).<br>(56) I have realised that, here in Norway, no matter how older people are, they want to do everything they can. So yes, here, autonomy is encouraged a lot. Is it always fulfilled? No, but you try (no. 14).<br>(57) In Norway, older patients are left in the hands of professionals when they really could have a more significant role in their care (no. 15).<br>(58) With those most cooperative, I have tried to get older patients to do things too. Others don't because there's no way for them to collaborate, and you get to know them (no. 15).
**Major theme 4: Lack of organisation, time and staff**	
**Minor theme 7: "Subservient to when they come"**	**Minor theme 8: Extremely short on time and understaffed**
**Norwegian older patients**	**Norwegian older patients**
(59) They usually tell me when they are coming. Some arrange for them to come earlier, but sometimes they come a little late, and I call to see if they are coming. But mostly, they come (no. 1).<br>(60) There was one time they forgot about me, and I was pretty surprised. And it's not normal. They had just forgotten about me. It was an afternoon, yes. And then they worried me (no. 1).<br>(61) They come directly. And if I'm not at home, they put the medicines inside the house (no. 2).<br>(62) They come when it suits them… It works for me, too (no. 3).<br>(63) They come when it is best for them to arrive. It's tough. After all, I want to have an apparent time reference because otherwise, I can't go out, and I have to sit and wait for them to come. Otherwise, I could go out between hours and be prepared if I knew when they were coming. And that's important because I was used to going out whenever I wanted, but for a while now, I've been sitting at home a lot, and it doesn't seem right to me (no. 4).	(70) They have very little time, but some are skilful at making time. Because they go to so many places and so many medicines for us... (no. 1).<br>(71) They may be swamped (no. 3).<br>(72) Yes, there have been times when those who have come had three patients simultaneously. That is not possible. They had two in an outlying area and one near here. And can they come to me at 8 a.m.? It's not just about me, but also about them (no. 4).<br>(73) They are swamped! Some stand up and don't sit down to give me the medicines, and I get nervous; you could say because I understand they are very busy. And there have been times when I was in much pain, and they didn't even notice [she laughs resignedly] (no. 4).<br>(74) No, think about time and patients. One, two, three, four, five, six, seven... And I am one more number among them (no. 7).<br>(75) They will have a lot to do, I suppose? Because they have come several times at 9:30 in the morning to give me the medicines, and it is a bit late, I think (no. 8).

(64) I'm subservient to when they come (no. 4).<br>(65) They come when they do best according to the patient list (no. 7).<br>(66) They usually come too late (no. 7).<br>(67) No, no, they come when they want (no. 8).<br>(68) They don't always come when they should, I wait here [at home], and there are times when they don't come, and I have to call them (no. 9).<br>(69) When it was 9 a.m., for example, they had yet to come, and I was going to have breakfast, and I had to wait until 10 a.m. (no. 11).	(76) There are few staff, few people. Yes, because there have been several who have left, and two young people come to cover them, and they don't always come knowing how things are and also, yes, it is not so easy to go and adapt so quickly (no. 9).<br>(77) Don't know. Do they forget… they don't have time…? That's what they usually answer. The reason will be that they don't have the capacity, they don't have time... (no. 9).<br>(78) Many others are more important than me because I only have compression stockings (no. 9).<br>(79) They don't have time, so you can't always count on them: lots to do, few people (no. 10).<br>(80) They have had difficulties because they need more staff. So…it's been tricky with the times they've come here, but okay (no. 11).<br>(81) Hire more people and pay them a better salary. That's… that's my suggestion. Because many of us are dependent, but... that's the way things are. They're very few. And when someone gets sick, it's almost a crisis (no. 11).
**Minor theme 9: "We try… But generally, it´s impossible"**	**Minor theme 10: Extremely short on time? and understaffed**
**Spanish migrant nurses**	**Spanish migrant nurses**
(82) [Are the visits adapted to the patients' routine?] We try; that's why there are no fixed visits every single day. It is asked in advance what time slot it´s best and if they have something to do, as likely as possible (no. 12).<br>(83) It is angrier when you go to a house, and the patient is not there. That's what the phone is for, to say you're not going to be there (no. 12).<br>(84) We can adapt to the patients' wishes at some specific moment. But generally, it´s impossible to follow all the opinions or needs. Because at the organisational level, it´s not possible (no. 14).<br>(85) We try to meet patients' preferences regarding hours. But then you must readjust because some still prefer a time slot, corresponding to another person needing it more (no. 15).	(86) We have very little time; it is pretty controlled. The time is short. Lack of staff makes us minimise visits (no. 12).<br>(87) I think that more [time] will always be needed, but I believe I give them the time they need (no. 12).<br>(88) Even tho I think that here [in Norway], we have more time than in Spain (no. 12).<br>(89) Sometimes, even you have to say, "I'm pretty pressed for time." And that's not the best thing (no. 12).<br>(90) I like treating patients and chatting with them while I do things. That's why I'm almost always late with my visit list [laughs] (no. 12).<br>(91) Lack of personnel, and that affects the patient. They are less listened to, or things could be done better. But that happens here and everywhere (no. 12).<br>(92) I have also worked in hospitals, and we don't have much time either (no. 13).<br>(93) I think older patients get the needed time. [pause] In my workplace, I believe that staff who don't have enough time it´s because they don't want to organise their time better or their free time, to dedicate to an older person (no. 14).
**Major theme 5: Consequences of lack of organisation, time and staff**	

Minor theme 11: Unsatisfied wishes	Minor theme 12: Mistrust in care situations
**Norwegian older patients**	**Norwegian older patients**
(94) Maybe I could use a bit more help (no. 2).<br>(95) I would always like to receive the same person. Or most of the time. Because continually explaining what they should do or how something should be done is tiring (no. 4).<br>(96) I´d probably need more help in the winter than in the summer due to my phases of depression (no. 7).<br>(97) It should be possible to agree on some time other than the scheduled visit because sometimes… [pause] I need someone who can "kick my a**". You know what I mean? And that they do not. Because I need… [gestures with hands] let's go! (no. 7).<br>(98) Perhaps they could offer to manage things for me about the doctor, the dentist, uh… Like those things that are so difficult for me to do alone. I am also anxious because of the oxygen I put on (no. 8).<br>(99) They could ideally use more time [laughs]. I have a shower daily; they only help me with a few things like changing my underwear and a compress because I don't wear a diaper, and uh… I could think about those kinds of little things (no. 8).	(100) Some don't know precisely what to do when they come, and that... And some fool around a bit... They don't turn the key when they leave (no. 4).<br>(101) The issue of alarms doesn´t work well. I called an ambulance. And then, the ambulance should have contacted the nurses earlier to get into my house because they [the ambulance´s staff] were waiting outside until a nurse came to open the door for them. So… I wish there was a better routine because we, the dependents, must have confidence. But I'm not very confident (no. 8).<br>(102) Some know more or can do more than others, so that [opportunity to choose who comes to her home] would be fine. But I'm not in that position; they go and do what they do (no. 9).<br>(103) With my BiPAP, the device. Everyone knows you have to wash it sometimes. Yes, it has to be done, and sometimes they forget. Some are very good, but others… (no. 8).<br>(104) They use the time they need but sometimes rush to finish (no. 9).<br>(105) It depends a bit on who is coming. Perhaps there have been some who have gone very fast. But I understand because maybe there was someone they had to go somewhere else, so… (no. 11).
**Minor theme 13: Imbalance in fulfilment of needs**	**Minor theme 14: Insecurity and mistrust feeling in the work context**
**Spanish migrant nurses**	**Spanish migrant nurses**
(106) Some have even asked me to take the dog for a walk. And I said, "no, sorry, my job is to help you, not take your dog out." And I have done things that some have asked of me even if I didn't feel like it (no. 12).<br>(107) You need more time to meet those extra requests. We complain about it (no. 12).<br>(108) They always need psychological support in the sense of human warmth. I think most patients need it, but you can't give it to them because you have to go. That´s the problem of quality of care (no. 12).<br>(109) I think patients do not receive what they need—for example, patients who have gone to the hospital due to an infection and are sent back home. And two days later, they had to be sent back to the hospital with sepsis because they were not given intravenous treatment (no. 14).	(113) If I'm not used to a patient, I need three times as much time as that colleague who is (no. 12).<br>(114) I have had a few instances where other colleagues have let me pass the buck. I think it's because they don't have the drive like us or feel insecure when making decisions. For example, I had a patient with significantly altered vital signs, and my colleagues did not see it as clear to notify the ambulance, so they asked me what to do (no. 15).<br>(115) I feel somewhat insecure when I have to trust some colleagues because I don't see them as safe regarding nursing competencies. I have had colleagues with years of experience who didn´t know how to provide intravenous treatment (no. 15).

(110) If there are cases in which tasks are evaded, I don't know if the reason is that they are older people or because nurses are saturated with work—or having certain patients with annoying personalities. In my case, there is an alcoholic patient who drinks a lot, and I don't feel like spending more time there than necessary (no. 14).<br>(111) In the emotional sense, if we believe that in Spain, the older people feel lonely, in Norway, they are double. The family support is much less, and that you spend twenty minutes in the morning with them is not going to meet their need. Because then they are isolated seven days a week (no. 15).<br>(112) There are situations where I think patients receive things they do not need. For example, an older person who lives alone at home has the right to receive something, and we go to their house to give them that. But it's more of a watchfulness that their children want, and maybe you're going to heat the food in the microwave, but it's something that person can do perfectly—a little for supervision or emotional support (no. 15).	
**Major theme 6: Cultural issues in the migration experience**	
**Minor theme 15: Perceptions and context in the migration experience**	
**Spanish migrant nurses**	
(116) Personally, I have adapted, but I don’t think I will ever fully adapt. I love my family a lot. I have my partner and son here; we have bought a house and plan to stay for several years but want to return to Spain (no. 12).<br>(117) [Regarding working conditions] I wouldn’t want to go back [laughs]. That’s the thing. For this reason, I am thinking about it, but my idea is to do a master’s degree in education to go into teaching. Because I love nursing, but when you have a family... Schedules are essential with my son (no. 12).<br>(118) I have earned much money because I have worked morning, afternoon and night shifts. Mainly my contract is for night shifts and some afternoons, and I will have to change it (no. 12).<br>(119) I would not like to return to Spain to work as a nurse. Because I don’t want to be called to work for two months, my contract runs out; they call me for another month, and my contract runs out... No. I instead work in the private sector or as a teacher (no. 12).<br>(120) Here is a much quieter life. [pause] My partner and I need to interact more with Norwegian people, but our group is more Spanish [laughs] (no. 12).<br>(121) It was difficult for me to adapt to meals and daylight hours in the beginning. I have lived in the north of Norway, and I have come to have only one hour of light a day. Psychologically it affects. I’ve been sad and tired (no. 13).<br>(122) I had an incorrect perception of what a Norwegian was. They are pretty open people and have never closed the door to me about anything. But the culture is entirely different from Spanish (no. 14).	
**Minor theme 16: Moments of discrimination in working places**	
**Spanish migrant nurses**	

(123) There are occasions that I have experienced when some older patients have preferred to speak with Norwegians because of the language. “Can you pass the phone to another nurse because you don't understand me?” (no. 12).
(124) There have been times when I have even wept because I felt attacked by someone. But that was a one-time thing (no. 12).
(125) I am an intense person; I speak loud. Some have told me to calm down [laughs]. But I don't know if they put a good face on me, but then they think something else negative. Anyway, they treat me well. So I don't care (no. 12).
(126) Anyways, I think that the Spanish are pretty well seen here. Beyond the stereotypes I have been told about “siestas”, I have been told that we are usually very hard-working and typically get our work done well (no. 12).
(127) I think that here the nurses get overwhelmed very quickly; perhaps it is because they are not used to the workload that we have in Spain (no. 13).
(128) As far as I know about nursing training, we are very, very well trained. In Norway, they raffle us and all the complicated techniques. If there is a Spanish nurse, they give them to her (no. 13).
(129) Once, they told me that a feeding pump had to be programmed for a patient, and a nurse jokingly told me that she had heard that Spanish nurses were very good at that [laughs]. “I make you a proposal, you program the pump, and I keep the phone and call the patients.” (no. 13).
(130) I have not had a problem with anyone, but I have heard other Spanish colleagues say that they have had some racist problems with other nurses. Although not that much in reality, many Norwegians travel a lot to Spain or have houses there (no. 13).
(131) In general, they [Norwegian people] have made me feel like one of the team. Except in one place where I worked, which created anxiety because my colleagues were not good to me. Because you are a foreigner or you are new to the workplace. I have felt somewhat discriminated against (no. 13).
(132) I have been told that, according to other colleagues, some patients preferred other nurses based on skin colour (no. 14).
(133) I have noticed that healthcare academic training here is lesser than we have in Spain. Here we, the Spanish nurses, are appreciated for our knowledge and training (no. 14).
(134) It has also happened that I have been with a colleague who didn´t want to explain some things to me because of the language, and they entrusted specific tasks to others (no. 15).
(135) I miss more communication between colleagues. Here they go more to the basics; in Spain, we sometimes give unnecessary details (no. 15).
(136) Being Spanish, I may have felt a little forced to do something in some situations. Since I was a Spanish nurse, I was skilful, so I did things when it was a task for another colleague (no. 15).
