# Supplementary material for: Analysing Power Relations among Older Norwegian Patients and Spanish Migrant Nurses in Home Nursing Care: A Critical Discourse Analysis Approach from a Transcultural Perspective
Source: Healthcare (Basel). 2023 Apr 29;11(9):1282. doi: 10.3390/healthcare11091282 (PMC10178409; doi:10.3390/healthcare11091282)
Supplement: Supplementary file 1 [file healthcare-11-01282-s001.zip › healthcare-2287073-supplementary/230301_Table-S4-magnitudes.pdf]

Table S4. Magnitude of the derived qualitative findings.

	Major themes (magnitude in %)	Minor themes (magnitude in %)	Speech acts (magnitude in %)	Pattern A Major themes (magnitude in %)	Pattern A Minor themes (magnitude in %)	Pattern A Speech acts (magnitude in %)	Pattern B Major themes (magnitude in %)	Pattern B Minor themes (magnitude in %)	Pattern B Speech acts (magnitude in %)	Pattern C Major themes (magnitude in %)	Pattern C Minor themes (magnitude in %)	Pattern C Speech acts (magnitude in %)
Norwegian older patients	-	7 (43,8%)	69 (50,7%)	-	3 (18,75%)	34 (25%)	-	4 (25%)	35 (25,7%)	-	0	0
Spanish migrant nurses	-	9 (56,2%)	67 (49,3%)	-	3 (18,75%)	24 (17,6%)	-	4 (25%)	22 (16,2%)	-	2 (12,5%)	21 (15,5%)
In total	6 (100%)	16 (100%)	136 (100%)	3 (50%)	6 (37,5%)	58 (42,6%)	2 (33,3%)	8 (50%)	57 (41,9%)	1 (16,7%)	2 (12,5%)	21 (15,5%)
