# Supplementary material for: Analysing Power Relations among Older Norwegian Patients and Spanish Migrant Nurses in Home Nursing Care: A Critical Discourse Analysis Approach from a Transcultural Perspective
Source: Healthcare (Basel). 2023 Apr 29;11(9):1282. doi: 10.3390/healthcare11091282 (PMC10178409; doi:10.3390/healthcare11091282)
Supplement: Supplementary file 1 [file healthcare-11-01282-s001.zip › healthcare-2287073-supplementary/230406_Scheme-S1-booklet-norwegian-patients.pdf]

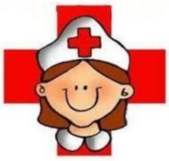

«Therapeutic collaboration in primary care for older people. An approach through critical discourse analysis»

This short presentation will give you some information about what we would like to investigate together...

The primary purpose of this research is to see if the attention the older patients receive is due to various considerations related to old age (or being older). This will be assessed based on autonomy and decision-making...

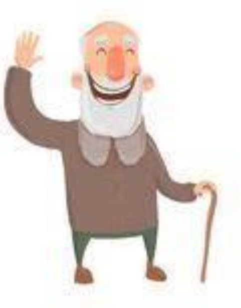

In other words: If older people are allowed to decide for themselves, or choose to do something, because they themselves believe they can do it. We want to investigate whether the older people are allowed to do this, or whether prejudice and other people's thoughts set limitations for the older patients.

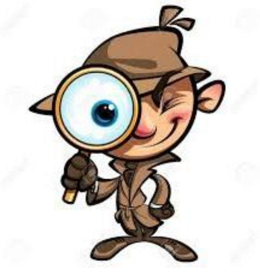

The answers to this can be discovered through interviews and you have the opportunity to participate in an interview within a few days. The interview will last approx. 30 minutes (exact time spent will depend on each individual interview). The questions will be simply formulated and you are free to answer them however you wish. You can withdraw from the study at any time.

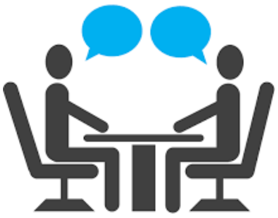

Thank you very much for your time and participation!

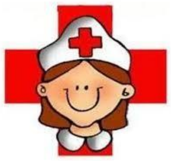

«Terapeutisk samarbeid i primæromsorgen for eldre menneske. En tilnærming gjennom kritisk diskursanalyse»

Gjennom denne korte presentasjonen vil du få litt informasjon om hva vi ønsker å gjøre sammen med deg...

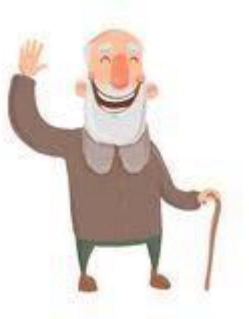

Hovedformålet med denne undersøkelsen er å se om oppmerksomheten eldre får skyldes ulike hensyn relatert til alderdom (evt. det å være eldre). Dette vil bli vurdert ut fra autonomi og beslutningstaking...

Med andre ord: Om mennesker får lov til å bestemme over selv, eller velge å gjøre noe, fordi de selv tror de kan gjøre det. Vi ønsker å undersøke om eldre får lov til det å gjøre dette, eller om fordommer og andre menneskers tanker setter begrensninger for de eldre.

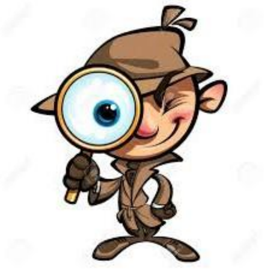

Svarene på dette kan bli oppdaget gjennom intervjuer og du har muligheten til å delta på et intervju innen få dager. Intervjuet vil vare ca. 30 minutter (eksakt tidsbruk vil avhenge av hvert enkelt intervju). Spørsmålene vil være enkelt formulert og du har frihet til å besvare dem slik du måtte ønske. Du kan når som helst trekke deg fra studien.

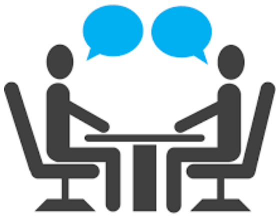

Tusen takk for din tid og deltakelse!
