# Supplementary material for: Analysing Power Relations among Older Norwegian Patients and Spanish Migrant Nurses in Home Nursing Care: A Critical Discourse Analysis Approach from a Transcultural Perspective
Source: Healthcare (Basel). 2023 Apr 29;11(9):1282. doi: 10.3390/healthcare11091282 (PMC10178409; doi:10.3390/healthcare11091282)
Supplement: Supplementary file 1 [file healthcare-11-01282-s001.zip › healthcare-2287073-supplementary/230406_Scheme-S2-a-b-interview-guide.pdf]

Scheme S2a. Interview script for Norwegian older patients.

Preliminary questions

1. How has your experience with nursing home care been?

2. Do you think they somehow treat you differently because of your age?

    a. [Yes] In which terms?

    b. [No] Why not? **Reasons**.

Appointments

3. When arranging an appointment, do nurses take you into account to establish the time slot for visits? [*care organisation*]

Nursing care services

4. Do you think the nursing care services you receive are those you really need? [*convenience*]

    a. [Yes] Do you receive those nursing care services when you need them? [*timely*]

    b. [No] Why not? **Reasons**.

5. Have you expressed your opinion regarding the nursing care services you receive or the treatment you take?

    a. [Yes] In which situations?

    b. [No] Why not? **Reasons**.

Nursing home visits

1. Do the nursing home visits adapt to your daily life/ lifestyle? [*balance*]

2. Do you consider the nurses to spend the necessary time you need with you? [*dedication*]

    a. In case of not: **Reasons**.

3. Do the nurses attend to any extra tasks you ask them in case of need?

    a. In case of not: **Reasons**.

Preferences expression and shared decision-making situations

1. Have you chosen something or decided something regarding your care?

    a. [Yes] In which situations?

        i. Have you been able to do that the way you wanted to? [*coincidence*]

    b. [No] Why not? **Reasons**.

Active participation situations

1. What is for you "to engage in your care"?
2. Do you think you engage as a patient in the nursing care services you receive?

    a. [Yes] In which situations?

        a. Did you want to engage then? And in that way? [*coincidence*]

    b. [No] Why not? **Reasons**.

Professional behaviours

1. When you have any preference or think about something related to your health condition: how is it to tell the nurses?

    a. [If easy] How do you do it?

    b. [If difficult] Why? **Reasons**.

2. Do you think the nurses listen to you if you want to share something with them?

    a. [Yes] Do the nurses follow your decision to be made?

    b. [No] **Reasons**.

3. Do the nurses do everything for you or encourage you to do something regarding your care? [*taking advantage of capabilities*]

Scheme S2b. Interview script for Spanish migrant nurses.

Preliminary questions

1. How has your experience with nursing home care been?
2. Do you think you somehow treat older patients differently?
    a. [Yes] In which terms?
    b. [No] Why not? **Reasons**.

Appointments

3. When arranging an appointment, do you consider the older patient to establish the time slot for visits? [*care organisation*]

Nursing care services

4. Do you think the nursing care services you give are those the older patients really need? [*convenience*]
    a. [Yes] Do you think you give those nursing care services when they need them? [*timely*]
    b. [No] Why not? **Reasons**.
5. Have you allowed the older person to give their opinion regarding the nursing care services you provide or the treatment they receive?
    a. [Yes] In which situations?
    b. [No] Why not? **Reasons**.

Nursing home visits

6. Do nursing home visits adapt to older patients´ daily life/lifestyles? [*balance*]
7. Do you consider you spend the necessary time older patients need with them? [*dedication*]
    a. In case of not: **Reasons**.
8. Do you attend to any extra tasks older patients ask you in case of need?
    a. In case of not: **Reasons**.

Preferences expression and shared decision-making situations

9. Have you allowed older patients to choose or decide about their care?

    c. [Yes] In which situations?

        i. Have they been able to do that the way they wanted to? [*coincidence*]

    d. [No] Why not? **Reasons**.

Active participation situations

10. What is for you "to engage in care"?

11. Do you think older patients engage in your nursing care services?

    c. [Yes] In which situations?

        a. Do you think they wanted to engage then? And in that way? [*coincidence*]

    d. [No] Why not? **Reasons**.

Professional behaviours

12. How do you think it is for older patients to tell you something?

    a. [If easy] How do they do it?

    b. [If difficult] Why? **Reasons**.

13. Do you think you listen to older patients when they want to share something with you?

    a. [Yes] Do you follow their decision to be made?

    b. [No] **Reasons**.

14. Do you do everything for the older patient or encourage them to do something regarding their care? [*taking advantage of capabilities*]

Cultural experience

15. From a cultural point of view, what has this experience meant to you? [personal/ work level]

16. Do you think that the fact of being Spanish has influenced something in this experience?

    a. [Yes] **Reasons.**

    b. [No] **Reasons**.
